# Toward Exosome-Based Therapeutics: Isolation, Heterogeneity, and Fit-for-Purpose Potency

**DOI:** 10.3389/fcvm.2017.00063

**Published:** 2017-10-09

**Authors:** Gareth R. Willis, Stella Kourembanas, S. Alex Mitsialis

**Affiliations:** ^1^Division of Newborn Medicine, Department of Medicine, Boston Children’s Hospital, Boston, MA, United States; ^2^Department of Pediatrics, Harvard Medical School, Boston, MA, United States

**Keywords:** exosomes, extracellular vesicles, exosome-based therapeutics, mesenchymal stem cells, preclinical

## Abstract

Exosomes are defined as submicron (30–150 nm), lipid bilayer-enclosed extracellular vesicles (EVs), specifically generated by the late endosomal compartment through fusion of multivesicular bodies with the plasma membrane. Produced by almost all cells, exosomes were originally considered to represent just a mechanism for jettisoning unwanted cellular moieties. Although this may be a major function in most cells, evolution has recruited the endosomal membrane-sorting pathway to duties beyond mere garbage disposal, one of the most notable examples being its cooption by retroviruses for the generation of Trojan virions. It is, therefore, tempting to speculate that certain cell types have evolved an exosome subclass active in intracellular communication. We term this EV subclass “signalosomes” and define them as exosomes that are produced by the “signaling” cells upon specific physiological or environmental cues and harbor cargo capable of modulating the programming of recipient cells. Our recent studies have established that signalosomes released by mesenchymal stem/stromal cells (MSCs) represent the main vector of MSC immunomodulation and therapeutic action in animal models of lung disease. The efficacy of MSC-exosome treatments in a number of preclinical models of cardiovascular and pulmonary disease supports the promise of application of exosome-based therapeutics across a wide range of pathologies within the near future. However, the full realization of exosome therapeutic potential has been hampered by the absence of standardization in EV isolation, and procedures for purification of signalosomes from the main exosome population. This is mainly due to immature methodologies for exosome isolation and characterization and our incomplete understanding of the specific characteristics and molecular composition of signalosomes. In addition, difficulties in defining metrics for potency of exosome preparations and the challenges of industrial scale-up and good manufacturing practice compliance have complicated smooth and timely transition to clinical development. In this manuscript, we focus on cell culture conditions, exosome harvesting, dosage, and exosome potency, providing some empirical guidance and perspectives on the challenges in bringing exosome-based therapies to clinic.

## Introduction

**Figure 1:**
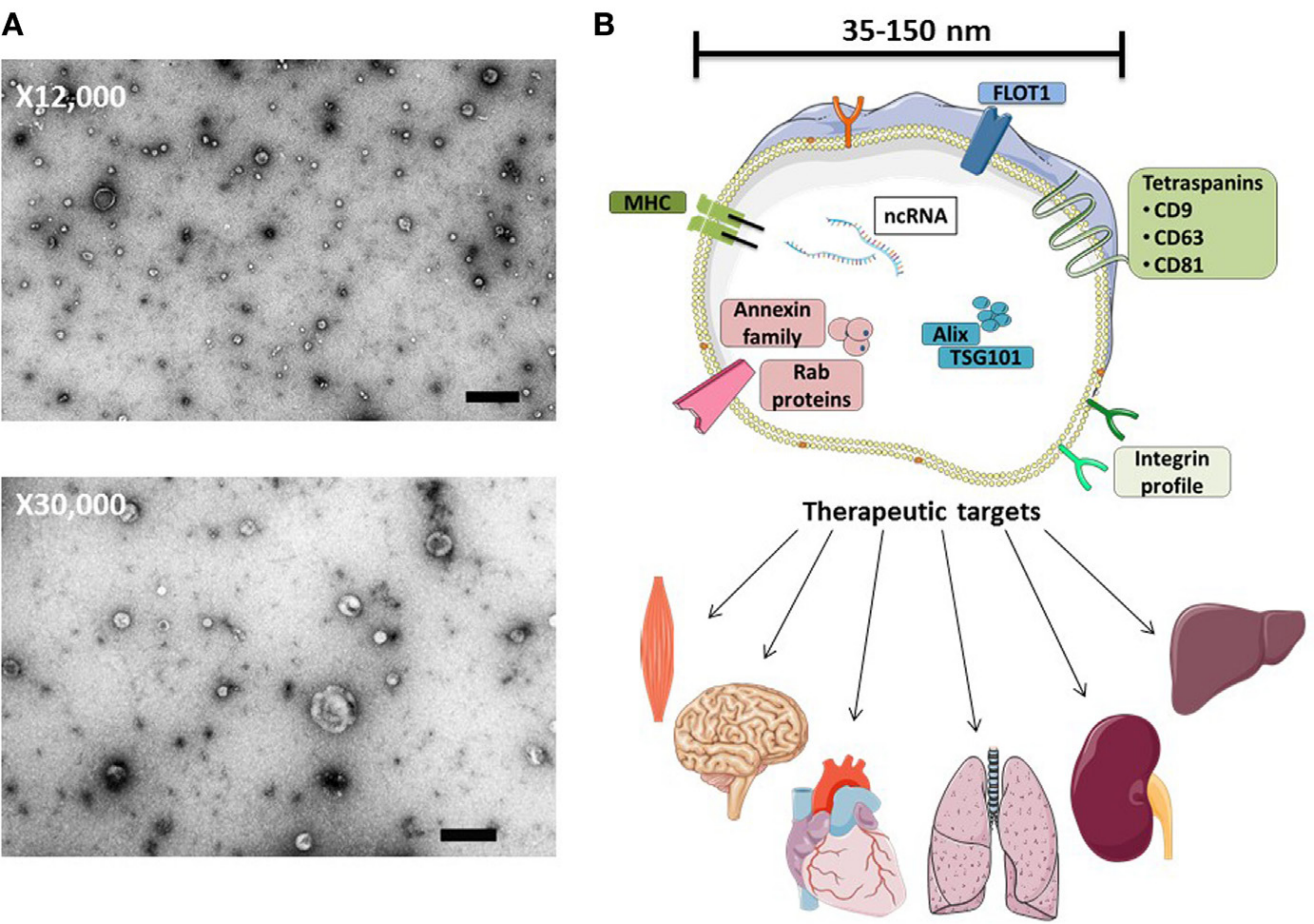
MSC-exosome morphology and composition. **(A)** Transmission electron microscopy (TEM) images of human bone marrow-derived MSC-exosomes (low magnification, 12,000×, scale bar = 500 nm, and high magnification, 30,000×, scale bar = 100 nm) representative TEM images adapted from Ref. (8). **(B)** MSC-exosomes are surrounded by a phospholipid bilayer and may contain proteins, such as annexins (these are important for transport); tetraspanins such as CD9, CD81, and CD63; and other proteins, such as Alix and TSG101, that are involved in exosomal biogenesis from endosomes. MSC-exosome therapy has shown beneficial effects in numerous preclinical models, demonstrating histological and functional benefits in multiple organs. Abbreviations: FLOT1, flotillin-1; MHC, major histocompatibility complex; TSG101, tumor susceptibility gene 101.

The intracellular transfer of diverse moieties *via* extracellular vesicles (EVs) has been proposed to be a widespread process. Cells release diverse EVs that include exosomes, microvesicles (MVs) and apoptotic bodies (1, 2). The classification of such EV subtypes is mainly based on their biogenesis and resultant biophysical properties, such as size, density, and predominant protein markers. Originally, the class of EVs generated through the endosomal pathway (exosomes) was assumed to represent a mere mechanism for the cell to jettison unwanted moieties (3, 4). We now understand that exosome biogenesis is a process governed by the endosomal-sorting complex machinery and involves the formation of intraluminal vesicles within multivesicular bodies (MVBs). Mature MVBs fuse with the plasma membrane and subsequently secrete the enclosed exosomes into the extracellular environment. During their biogenesis, exosomes associate with an array of bioactive cargo from their parental cell. Such cargo has been reported to include genetic information in the form of small noncoding RNAs, free fatty acids, surface receptors, and proteins (Figure 1) (5, 6). It is considered that the biophysical properties of EVs, including their cargo, reflect the stimulus triggering their formation (7), implying specific packaging of “*message*” prior to export from the parent cell. In turn, the secretion of these biologically loaded signaling vectors to the extracellular environment represents an important method of cell-to-cell communication, dubbed the “new endocrinology”.

Exosomes have been shown to play important roles in a broad range of pathological conditions, such as cancer (9), liver and kidney disease (10), neurodegenerative disorders (11), and numerous cardiopulmonary disorders (12, 13). More recently, in addition to their prognostic and diagnostic value, exosomes have also been reported to represent novel therapeutic reagents across multiple disciplines.

## Exosome-Based Therapeutics

The therapeutic capacity of exosomes generated by mesenchymal stem/stromal cells (MSCs) that have been derived from different organs, such as bone marrow, umbilical cord, adipose tissue, or placenta has been tested in various disease models. In the cases where cells and their respective exosomes were studied in parallel, exosome treatment has demonstrated a similar or even superior therapeutic capacity to MSC treatment (14). MSC-exosomes have provided beneficial effects in numerous disease models promoting functional recovery and neurovascular plasticity following traumatic brain injury (15), reducing myocardial infarction size (16, 17), ameliorating hypoxia-induced pulmonary hypertension (18), aiding repair of kidney injury (19, 20), and orchestrating neurological protection by the transfer of microRNA (21, 22). MSC-exosome-based approaches for the treatment of different disease models are highlighted in Table 1.

**Table 1:** Summary of MSC-exosome-based approaches for the treatment of different disease models.

Disease model	MSC-product “nomenclature”	Isolation method	Dose assessment	Dose	Reference
**Respiratory**					
Bronchopulmonary dysplasia	Exosomes	Density Cushion	Cell equivalent	0.5 × 10^6^	Willis et al. (8)
Pulmonary hypertension	Exosomes	SEC	Protein	0.1–10 μg	Lee et al. (18)
Pulmonary hypertension	Exosomes	UC (100K × *g*)	Protein	25 μg	Aliotta et al. (23)
Acute lung injury	Microvesicles (MVs)	UC (100K × *g*)	Cell equivalent	1.5 × 10^6^	Zhu et al. (24)
Silicosis	Exosomes	Sucrose gradient	Protein	40 μg	Phinney et al. (25)
Pneumonia	MVs	UC (100K × *g*)	Cell equivalent	9 × 10^6^	Monsel et al. (26)

**Cardiovascular**					
Myocardial infarction	Exosomes	ExoQuick	Cell equivalent	4 × 10^6^	Yu et al. (27)
Myocardial infarction	Extracellular vesicles (EVs)	UC (100K × *g*)	Protein	80 μg	Bian et al. (28)
Myocardial infarction	Exosomes	ExoQuick	Protein	80 μg	Teng et al. (29)
Ischemia/reperfusion	Exosomes	HPLC	Protein	0.4 μg	Lai et al. (16)
Ischemia/reperfusion	Exosomes	HPLC	Protein	0.4–0.8 μg	Arslan et al. (17)

**Neurological**					
Traumatic brain injury	EVs	Anion exchange chromatograph	Protein	30 μg	Kim et al. (30)
Laser-induced retinal injury	Exosomes	UC (110K × *g*)	Protein	10 μg	Yu et al. (31)
Optical nerve crush	Exosomes	UC (100K × *g*)	ExoELISA	3 × 10^9^	Mead and Tomarev (32)
Stroke	EVs	UC (110K × *g*)	Cell equivalent	2 × 10^6^	Doeppner et al. (33)
Stroke	Exosomes	UC (100K × *g*)	Protein	100 μg	Xin et al. (34)

**Musculoskeletal**					
Cardiotoxin injury	EVs	UC (100K × *g*)	Protein	5 μg	Lo Sicco et al. (35)

**Hepatic**					
Drug-induced liver injury	Exosomes	UC (100K × *g*)	Protein	0.4 μg	Tan et al. (36)
Liver fibrosis	Exosomes	UC (100K × *g*)	Protein	250 μg	Li et al. (37)

**Gastrointestinal**					
Colitis	EVs	UC (100K × *g*)	Protein	50–200 μg	Yang et al. (38); Fang et al. (39)

**Dermatological**					
Wound healing	Exosomes	UC (100K × *g*)	Protein	160 μg	Zhang et al. (40)
Wound healing	Exosomes	UC (120K × *g*)	Protein	100 μg	Fang et al. (39)

**Renal**					
Ischemia/reperfusion	MVs	UC (100K × *g*)	Protein	100 μg	Zou et al. (41)
Acute kidney injury	MVs	UC (100K × *g*)	Protein	100 μg	Bruno et al. (42)

*Ultracentrifugation (UC): 100,000–120,000 × *g* (100K–120K × *g*). Size-exclusion chromatography (SEC). ExoQuick and ExoELISA refer to a commercially available exosome isolation kit and CD63 capture (exosome) ELISA, respectively (Systems Biosciences, CA, USA)*.

While the functional roles of exosomes have been extensively reported [reviewed in Ref. (43–46)], few reviews have addressed the challenges underlying the transition of exosome-based therapies from animal models to clinical development. Furthermore, the full realization of their therapeutic potential has been hampered by a lack of standardization in exosome isolation and characterization. Herein, we will focus on the therapeutic application of MSC-exosomes and outline topics relevant to the facilitation of their development as a pharmaceutical preparation, focusing on exosome harvesting, dosing and potency, and providing guidance on the current challenges in bringing exosome-based therapies to clinic.

## Mesenchymal Stem/Stromal Cell Origin: Where are the Exosomes Coming From?

A comprehensive characterization of the tissue/cellular source of exosomes is imperative for exosome-based therapeutics. Detailed methods for obtaining human MSCs from several tissues, including bone marrow (BMSCs), Wharton’s jelly (WJMSCs), umbilical cord blood, and adipose tissue are well reported (47, 48). By definition, MSCs must adhere to plastic, demonstrate a baseline differentiation potential to osteocytes, chondrocytes, and adipocytes *in vitro*, and express the presence of widely accepted surface markers (Table 2) (49). However, donor-to-donor variability remains a prominent challenge. Studies have found that BMSCs obtained from older donors have slower proliferation and reduced differentiation potential *in vitro*. Furthermore, discrepancies in the differentiation capacity and transcriptome profiles are reported to be tissue and species dependent (50–52). To what extent do these uncertainties affect the therapeutic capacity of MSCs and their resultant exosomes remains unclear. Thus, in addition to validated MSC isolation procedures, investigators should adhere to carefully selected donor eligibility criteria in accordance with the appropriate ethical and regulatory approval, employing strict control measures to prevent risk of relevant communicable disease agents or diseases (RCDADs), such as human immunodeficiency virus (HIV), hepatitis C virus, and cytomegalovirus. Moreover, donor screening should include a comprehensive medical record review, physical assessment, and medical history interview, with records documented in compliance with appropriate regulatory frame. The International Society of Extracellular Vesicles (ISEV), the Food and drug Administration (FDA), the International Council for Harmonization (ICH) of Technical Requirements for Pharmaceuticals for Human Use, and the European Medicines Agency (EMA) provide extensive guidance for the development and generation of novel biological medicines with regard to donor/patient care, product safety, and quality (53–56). The demonstration that exosomes generated by MSCs isolated from WJMSCs are as effective as BMSC-exosomes in treating rodent disease models (8) may facilitate standardization and consistency of MSC lines for exosome harvesting. Moreover, the umbilical cord may possess several advantages over bone marrow. First, the umbilical cord represents a more readily available source than bone marrow. Second, it is often viewed as discarded medical waste that does not require any invasive procedures or cadaver procurement.

**Table 2:** Minimal criteria for defining MSCs, as put forth by The International Society for Cellular Therapy.

Characterization of mesenchymal stem/stromal cells (MSCs)
Adherence to plastic in standard culture conditions Phenotype: Positive (≥95%) CD105 CD45 CD73 Negative (≤2%) CD34 CD90 CD14 or CD11b CD79a or CD19 HLA-DR *In vitro* differentiation: osteocytes, adipocytes, and chondrocytes.

*Human leukocyte antigen–antigen D related (HLA-DR). Adapted from Dominici et al. (49)*.

## “Physioxia” Considerations in Establishing MSC Culture Conditions for Exosome Production

Previous studies have reported that the protein and RNA profile of exosomes reflect the cell culture conditions and microenvironmental stimuli that triggered their release. With this in mind, it begs to question can “stimulating” and/or preconditioning cells be used as a means to generate a more homogenous or efficacious exosome population? Interestingly, in an experimental model of hyperoxia-induced bronchopulmonary dysplasia (BPD), Waszak and coworkers found that the conditioned media (CM) derived from hyperoxia-preconditioned rat BMSCs (95% O_2_, for 24 h) provided greater protection *in vivo* compared to CM collected from cells grown under control conditions (57). Clearly, in the absence of further characterization, one can only speculate where the observed augmentation of activity resides. Other studies have found that MSCs cultured in hypoxia conditions (<5% O_2_) exhibited an altered protein expression pattern compared to MSCs cultured in the so-called “normoxia” (58). Furthermore, in a murine hind limb ischemia model, they showed that intra-arterial injection of MSCs cultured in both “normoxic” or hypoxic conditions enhanced revascularization compared with saline controls; however, the functional recovery of mice that received hypoxia preconditioned MSCs was faster (59). These reports suggest that preconditioning MSCs in different oxygen environments may improve their tissue regenerative potential.

Indeed, “hypoxia” (1% O_2_) has been shown to increase exosome production in numerous cell types *in vitro*. Previous studies have shown that the hypoxia-induced elevation in exosome secretion is chiefly governed by hypoxia-inducible factor-1 alpha (HIF-1α) and is independent of apoptosis (60). Often, laboratory cell culture conditions are at atmospheric oxygen levels (21% O_2_, corresponding to a PO2 of ~159 mmHg); following adjustment to 5% CO_2_, this equates to ~19.95% O_2_ (~150 mmHg). Although, it is challenging to accurately measure tissue oxygen concentrations *in vivo*, it is well recognized that most of the human body tissue is normally exposed to much lower O_2_ levels. This can range from 160 to 100 mmHg in the alveoli, >35 mmHg in the brain, and ~25 mmHg in skeletal muscle (61–63). Notably, in the bone marrow, arguably the most common origin of MSCs, the PO_2_ is reported to be ~40 mmHg, while the umbilical cord vasculature PO2 is reported to be between 10 and 30 mmHg (63). Organ oxygen levels have been extensively reviewed (63). However, it is important to remember that the different techniques used to measure oxygen concentration *in vivo* are subject to their own advantages and limitations.

*In vivo*, the oxygen concentration of an organ is an indication of its physiological state and reflects the balance between oxygen delivery and its metabolic consumption. Consequently, in a physiological condition, organs are subject their own unique “physioxia” status. On balance, the routine laboratory cell culture conditions expose MSCs to oxygen levels higher than those in their physiologic niches, and this departure from “physioxia” may precipitate a “perceived hyperoxia” response. Thus, it is important to recognize this factor when interpreting results of experiments performed in atmospheric “normoxia,” and also to realize that the impact of this factor may vary, depending on the particular study and the metrics assessed.

In turn, several questions remain unanswered. The optimal oxygen concentration for *in vitro* MSC culture and the effect that it may have on subsequent exosome production remains undefined at this point. Existing reports indicate that optimization is likely to be both MSC origin and disease model specific. Thus, additional studies assessing the effect of oxygen levels on MSCs and their resultant exosomes are much needed.

## Exosome Heterogeneity

Cells generate three major EV classes: apoptotic bodies, MVs, and exosomes. Arguably, it is often assumed each subtype represents a homogenous vesicle population that can be distinguished based on biophysical properties such as size or density. However, it has become obvious that even within such subtypes, there is heterogeneity (2, 64). Although, the field lacks tools to distinguish vesicles from different routes of biogenesis, recent evidence has demonstrated that MSCs release distinct EV subpopulations that differ in biophysical, proteomic, and RNA repertoires. Specifically, Kowal and coworkers found that large-, medium-, and small-sized EVs can be isolated by sequential low-, intermediate-, and high-speed centrifugation, respectively. Among the small-EVs (exosomes), four subcategories were defined by their degree of enrichment in CD63, CD9, and/or CD81 tetraspanins (64). In accordance, Lai et al. found that MSCs secrete many distinct subtypes of vesicles, which differ in RNA and protein composition (65). It is relevant to note that the study involved an immortalized, iPS-derived MSC cell line that will likely secrete a more restricted range of exosome subtypes than those generated by primary cells.

**Figure 2:**
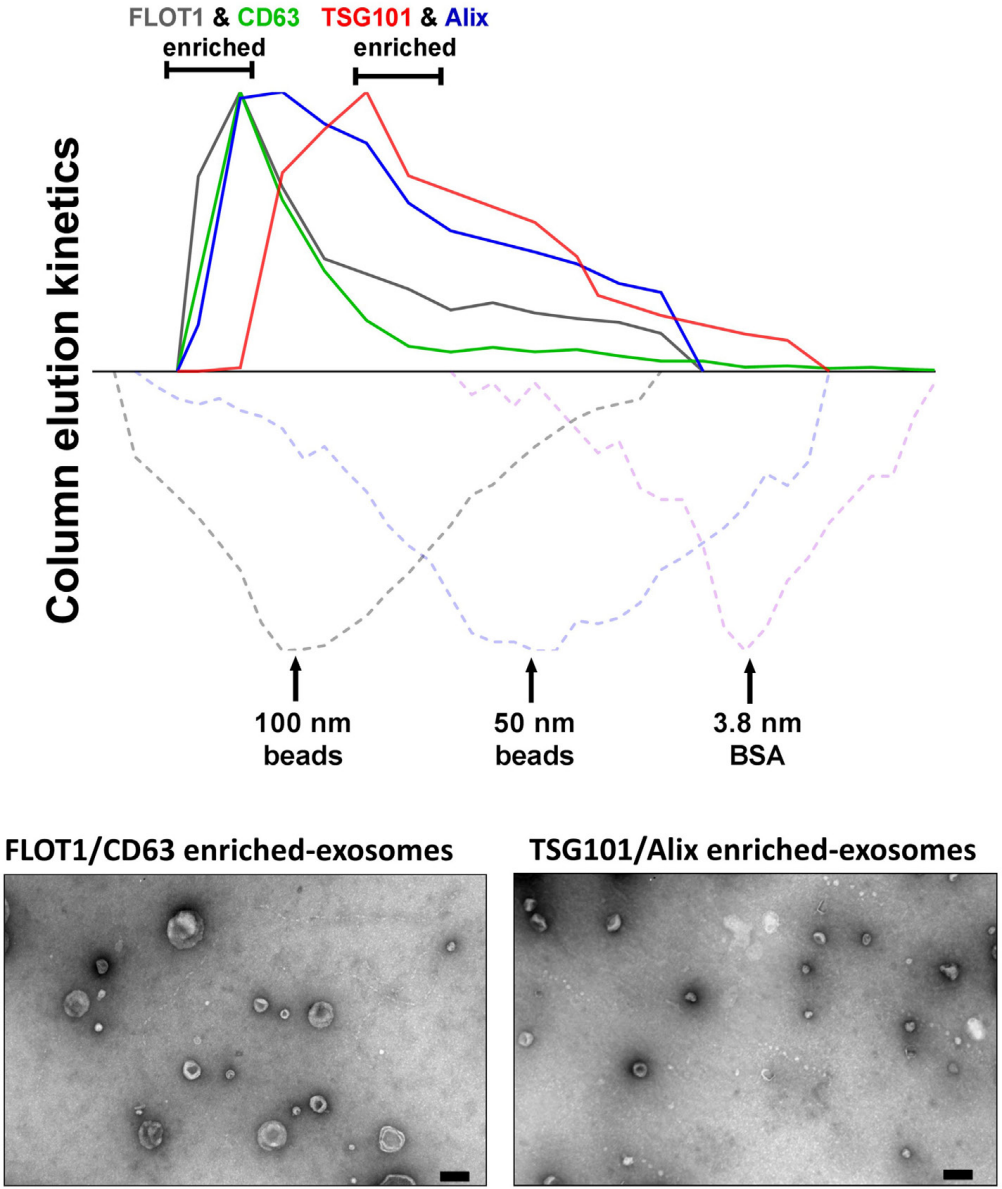
Isolation of MSC-exosomes subtypes by size-exclusion chromatography (SEC). Briefly, exosomes were isolated directly from cell culture supernatants following a 36 h harvest period in serum-free-media. Cell culture media were subjected to differential centrifugation, 300 × *g* for 10 min, followed by 3,000 × *g* for 10 min, and 13,000 × *g* for 30 min to remove any cells, cell debris, and large apoptotic bodies in suspension, respectively. Conditioned media (CM) was concentrated 50-fold by tangential flow filtration (TFF) and the exosomes were purified using OptiPrep™ (iodixanol) cushion density flotation (3.5 h at 100,000 × *g*, 4°C), as previously described (8). Heterogeneous exosomes were further purified by size using size-exclusion chromatography (SEC). Here, sepharose CL-2B (80 mL) was washed with 1 × SSPE buffer [containing 1 mM EDTA and 149 mM NaCl in 0.20 mM phosphate buffer (pH 7.4)]. The column was packed with washed sepharose CL-2B to create a column with an internal diameter of 1.6 cm and height of 40 cm. Exosomes (1 ml, corresponding to 60 × 10^6^ MSC equivalents) were added to column with a flow rate of 1 ml/min. Fractions (1 ml) were collected and assessed by dot plots and electron microscopy. The elution kinetics of 100 nm, 50 nm, and bovine serum albumin (BSA) were used to estimate exosome elution kinetics. TSG101, Alix, CD63, and FLOT1 levels were assessed by dot plots and are reported as relative intensity. Here, we identify two distinct MSC-exosome subtypes. The larger exosomes (>80 nm) have a greater flotillin-1 (FLOT1) and CD63 enrichment, while smaller exosomes (<80 nm) have a greater TSG101 and Alix ratio.

In our hands, ongoing studies aim to address the relationships between MSC-exosome subtypes and therapeutic efficacy and to explore the hypothesis that a discrete subtype is responsible for the therapeutic activity in our established experimental models of BPD, a chronic lung disorder of infants (8). Here, we isolated exosomes from either human BMSCs or WJMSCs by differential centrifugation, followed by tangential flow filtration and iodixanol density floatation before separating exosome subtypes by size-exclusion chromatography (SEC) (described in Figure 2). This approach separates MSC-exosome subtypes based on their size, and in accordance with previous reports, we demonstrate a shift in protein markers associated with different exosome subtypes. Specifically, we found that CD63 and flotillin-1 (FLOT1) is associated with “large”-exosomes (>80 nm), while Alix and TSG101 is enriched in “small”-exosomes (<80 nm). Few studies assess the ratio of such markers; however, further investigation is warranted as it may provide a tool for distinguishing exosome subtypes. Ongoing *in vivo* studies are testing whether the different MSC-exosome subtypes exhibit differential therapeutic efficacy in a number of animal models currently utilized by our group and collaborators.

Regulatory frameworks often require a mechanism-of-action that details the identity, quantification, and characterization of such bioactive substances responsible for the therapeutic effect(s). Moreover, disclosure of non-active components (“excipients”) in drug preparations should be acknowledged (56, 66). Knowing exosome subtypes harbor different protein and genetic cargo, it is fair to speculate that they likely mediate different effects on targets cells. Thus, improved separation techniques that distinguish between “non-active” and therapeutic exosome subtypes may help focus the search for the bioactive substance(s) responsible for such beneficial effects. The “one size fits all” hypothesis may not work for exosome-based therapeutics. Although a specific exosome subset may induce beneficial therapeutic effects in a specific disease model, it is important to recognize that a different exosome subpopulation may afford the beneficial effects in a different disease model.

## Emerging Themes in Exosome Isolation

Isolation methods impact exosome integrity, *in vivo* biodistribution and metabolic fate (56). Exosome isolation techniques from various biological fluids and cell culture medium have been extensively reviewed (67–69). It is well established that widely applied exosome isolation techniques, such as differential ultracentrifugation (UC), promote vesicle aggregation and often co-isolate soluble factors and protein (70). Thus, a consensus in the field has shifted toward more “*gentle”* isolation techniques to ultimately reduce contaminants (non-EV material), maintain integrity, and isolate “bioactive” vesicles from heterogeneous EV populations. To date, popular avenues of investigation include gradient density isolation and SEC, with the latter being more suited to enclosed tissues culture systems. Variations of such approaches have been shown to effectively separate exosomes from proteins and soluble factors in different biological fluids (71, 72). However, layered density-based procedures may achieve enrichment rather than true exosome isolation, where the influence of UC parameters coupled with high-sucrose concentrations may change the osmotic environment (69). Furthermore, UC methods are impracticable for large-scale bioprocessing.

**Figure 3:**
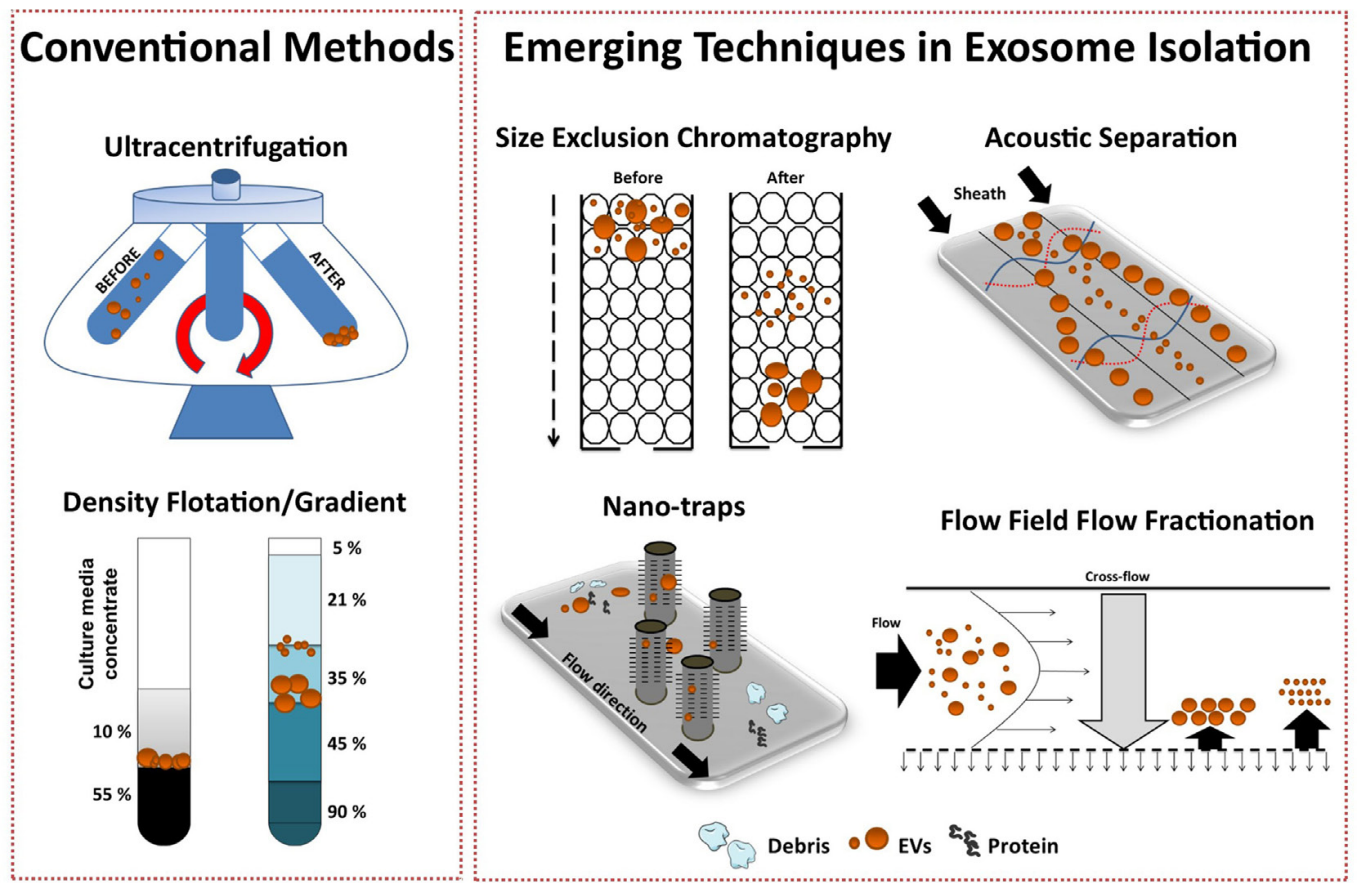
Conventional and emerging exosome isolation techniques. Ultracentrifugation (UC) is the most common exosome isolation method. Here, sedimentation of solutes including vesicles is governed by their size/density. Variations of UC such as layered or cushion based-density gradient UC are also widely employed. New methods are required to facilitate large-scale, high-yield production of exosomes for clinical applications. Several emerging technology platforms have shown promise in isolating exosomes from various sample matrices. Techniques, such as size-exclusion chromatography, ciliated micropillars nano-traps, acoustic wave separation technology, and flow field-flow fraction (F4), exploit unique biophysical traits of exosomes.

Several emerging technology platforms have shown promise in isolating exosomes from various sample matrices, with each method exploiting a particular biophysical trait of exosomes such as their size, density, shape, or surface receptors (Figure 3) (72). The final goal is an isolation method that is label free, distinguishes between exosome subtypes and interfering components, and can facilitate a large-scale production of exosomes.

Recently, Lee and colleagues employed a label-free acoustic nano-filter system to isolate exosomes based on their size (73). Specifically, using ultrasound standing waves exert differential acoustic force, they isolated exosomes from both cell culture media and stored red blood cell products. They effectively separated exosomes (149 nm) and MVs (410 nm). Although, its application for high-throughput exosome preparations is yet to be established, previous studies have used label-free acoustic wave systems to isolate circulating tumor cells (74).

Exosomes can also be separated by size with 10 nm accuracy using variations of flow field-flow fractionation (F4) (75). Asymmetrical F4 (AF4) is a one-phase chromatography method that uses parabolic-flow to drive exosomes across a flow channel. A crossflow intercepts the parabolic-flow perpendicular to the channel and distributes particulate components against the flow chamber wall. Subsequently, exosomes are separated based on differences in diffusivity. Smaller particles diffuse further from the accumulation wall and are eluted earlier than larger ones. Successful attempts have been made using AF4 to isolate exosomes from human neural stem cell culture (76) and melanoma cell lines (77). AF4 approaches provide promise for “label-free” isolation of large-scale exosome production.

To effectively utilize the size difference between exosomes, other EV subtypes and cellular debris, Wang and colleagues fabricated a porous silicon nanowire-on-micropillar “nano-trap” made from ciliated micropillars (78). This fabricated microfluidic device preferentially traps exosomes with a diameter of 40–100 nm, while filtering out proteins, larger EVs, and cellular debris. Moreover, trapped exosomes can be recovered by dissolving the porous silicon nanowires in PBS buffer. However, in this proof-of-concept study, the authors noted poor vesicle retention (~60%) and only assessed small sample volume (30 μl), thus scalability is yet to be demonstrated.

Overall, to support the development of exosome-based therapeutics, research efforts should focus on the development of “label-free” exosome isolation techniques that can support high-throughput systems/scale-up requirements and are capable of distinguishing exosome subtypes. Although a number of highly sophisticated technologies for EV isolation have emerged recently, their application is mainly in the biomarker field, as tools for exosome-based diagnostics. Although such emerging technologies hold great promise, the large-scale preparation of isolated exosome subtypes to be used as the basis of exosome-based pharmaceutical products will probably depend, at least in the near future, on modifications of classic industrial processes such as SEC.

## Dose Evaluation

Currently, investigators use several different methods to quantify exosome dosage, making inter-study comparison troublesome. Common quantitative practices include reporting cell equivalents, protein concentration, and/or specialized quantitative analytical measurements by instruments, such as tunable resistive pulse sensing (TRPS) and nanoparticle tracking analysis (NTA), with each method harboring its own advantages and limitations [for recent reviews (79–82)]. The need to standardize exosome dosing is imperative. Of interest, methods such as TRPS are currently used to verify particle size and characterization for liposome-encapsulated forms of doxorubicin and are accepted within the definition of bioequivalence as set forth by the FDA and EMA. Although enumeration of analytical criteria is beyond the scope of this review, we acknowledge that the field is limited by current technology and lacks the ability to accurately assess exosomes at a single vesicle level. Thus, to aid inter-study comparison, we recommend that in addition to providing extensive detail of standardized cell culture conditions and pre-analytical protocols, investigators should measure exosome concentration using multiple quantification tools, where possible. A summary of the advantages and limitations of common methods used to determine exosome dose are highlighted in Table 3. Establishing an exosome potency assay is a novel approach which holds great promise in standardizing exosome dosing.

**Table 3:** A summary of the advantages and limitations of common methods used to determine exosome dose.

Exosome dosing method	Information acquired	Advantages	Limitations
Protein	Total protein amount	Fast	May not reflect bioactive ingredient. May measure non-exosomal-associated protein. Does not reflect particle concentration/size/distribution
		Low cost	

Nanoparticle tracking analysis (NTA)	Particle concentration, size, and distribution (range 10 nm–2 μm)	Fluorescent-NTA available	Difficulties in determining vesicle aggregates and size heterogeneity in biological samples
		Provides absolute EV concentration, size and distribution	

Tunable resistive pulse sensing (TRPS)	Particle concentration, size and distribution (size range <40 nm–10 μm)	Provides absolute EV concentration, size and distribution. Can also measure particle surface charge	Different pore sizes are needed to assess biological samples that contain both exosomes (for example, <150 nm) and larger EVs (>150 nm)
			Can detect non-exosome material within size range

ELISA	Specific concentration of EV marker (for example, CD9 or CD63)	Specific to “exosome” protein	May not reflect bioactive exosome population. Time consuming. Provides non-specific information about exosome size/distribution

Dynamic light scattering (DLS)	Particle concentration and size (range <1 nm–10 μm)	Fast	Difficulties in measuring heterogeneous samples
		Small volume required	

Flow cytometry	Particles concentration and size (size range ~>150 nm)	Non-specialized (typical) laboratory equipment	Detection limit (~<150 nm) *(cytometer dependent)*
		Fluorescently labeled EVs	

Cell equivalents	Cell number	Low cost	Requires standardized tissue culture procedures. Does not reflect particle concentration/size/distribution
		Fast	

**Emerging tools for estimating exosome dose**	**Description**		

“Fingerprinting assays”	Quantifies surrogate markers (for example levels/ratios of exosome markers such as CD63, CD9, and CD81) as an indication of potency and/or dose		

Exosome potency assays	Quantifies the ability of an exosome preparation to elicit the desired biologic/therapeutic action or surrogate activity *in vitro* and/or *in vivo*		

*NTA and DLS size ranges were obtained from http://malvern.com. TRPS detection range was obtained from http://izon.com*.

## Developing an Exosome Potency Assay

**Figure 4:**
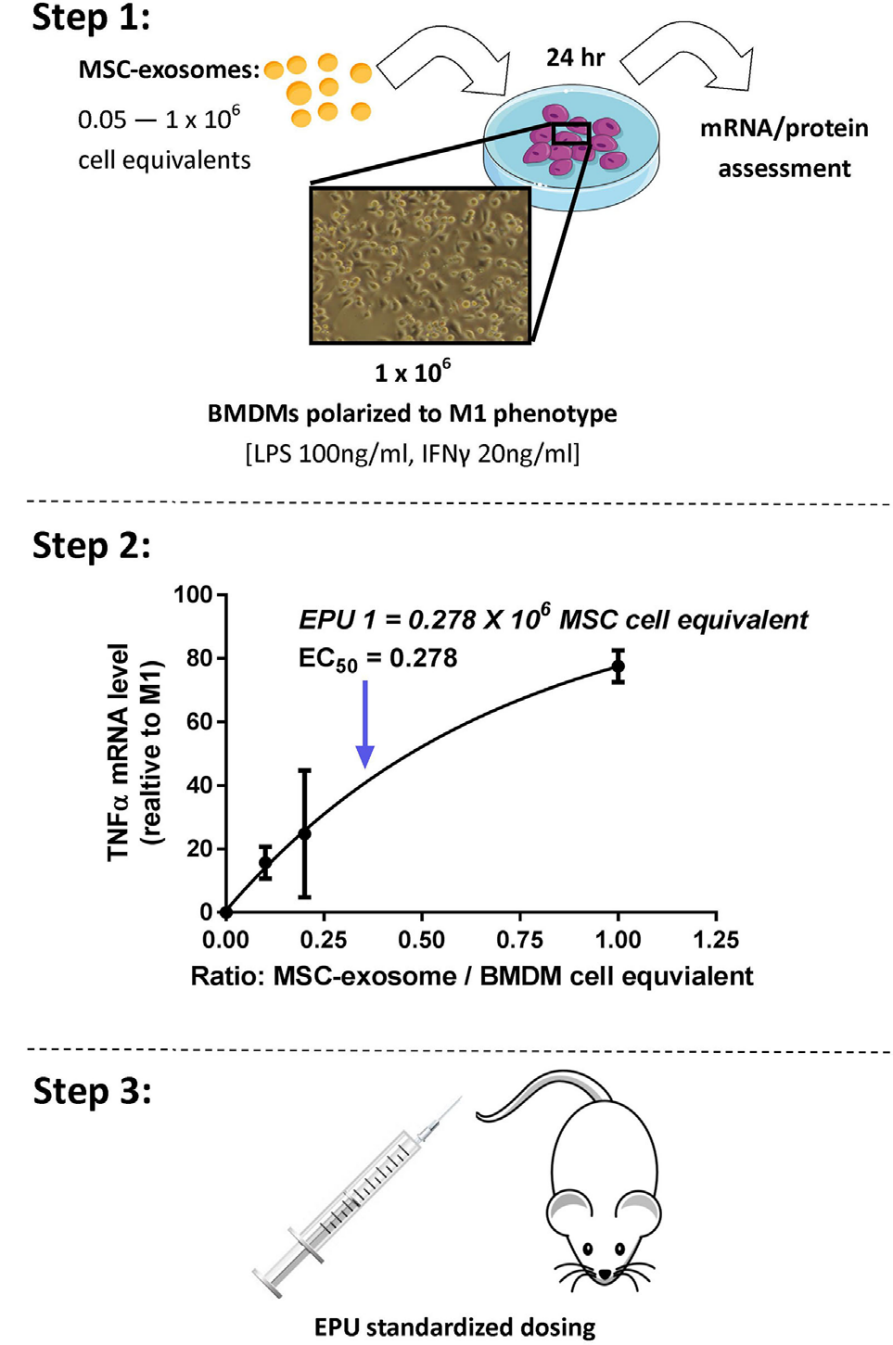
Stepwise approach to developing an MSC-exosome potency assay. Considering limitations in current exosome quantification techniques, assessment of exosome potency would be a valuable tool to standardize exosome dosing. Step 1: primary murine bone marrow-derived macrophages (BMDMs) were obtained by flushing the femur and tibia of 6- to 8-week-old FVB mice. M1 polarization was initiated by lipopolysaccharide (LPS) 100 ng/ml and interferon-γ (IFNγ) 20 ng/ml stimulation. Macrophage polarization (1 × 10^6^ BMDMs) was initiated with/without the presence of MSC-exosome preparations (0.05–1 × 10^6^ cell equivalents), providing several ratios of MSC-exosome cell equivalents-to-BMDMs. After 24 h, total RNA was isolated and TNFα mRNA levels were assessed by RT-qPCR. The functional endpoint is the capacity of MSC-exosomes to suppress the mRNA induction of TNFα. Step 2: an EC_50_ value (50% reduction in TNFα mRNA, relative to M1 control) is transformed to an arbitrary exosome potency unit (EPU). Data were adapted from our recent work (8), where we demonstrated purified human umbilical cord MSC-exosomes, dose dependently suppressed mRNA levels of TNFα in alveolar macrophages *in vitro*. Step 3: an EPU can be applied to standardize dosing between different exosome preparations or as a means of correlating potency to exosome quantity.

The definition of the bioactive substance(s) will remain a crucial question in the preclinical development of exosome-based therapeutics. With an orchestra of bioactive cargo and diverse physiological effects (Figure 1B), identification of “*one*” bioactive substance or a singular mechanism-of-action appears improbable. By FDA standards, potency is defined as the products specific ability or capacity to affect a given result (66). With no “gold-standard” technique for their quantification, assessment of exosome potency would be a valuable tool in overcoming the inconsistencies in preparations and batch-to-batch variation. For example, exosomes obtained from two separate donors may be normalized *via* a given quantitative method; however, the “*bioactive”* load may differ, subsequently the potency and degree of efficacy will not be the same. Thus, investigators should consider employing a unique exosome potency unit (EPU) to standardize practices and minimize variation between different samples. Presently, attempts to define an exosome potency metric utilized the immunomodulatory properties of MSC-exosomes. For example, Jiao et al. described an *in vitro* potency assay for MSC-exosomes based on the release of IL-10 from mononuclear cells following incubation with exosome preparations, and other studies have shown that T-cell proliferation assays may be modified to provide the basis for assays on exosome immunomodulatory potency (29, 83). Growing evidence also suggests that MSC-exosomes can modulate macrophage phenotypes (8, 35). Macrophages play a pivotal part in regulating immune responses. They assume both phagocytic “defensive” roles and exhibit regulatory “anti-inflammatory” actions, facilitating both the initiation and the resolution of inflammation (84). With this consideration in mind, our ongoing studies are exploring an *in vitro* macrophage polarization assay as a means of assessing MSC-exosome potency. Briefly, the potency assay involves adding MSC-exosomes to murine bone marrow-derived macrophages (BMDMs) that are polarized to the classically activated (proinflammatory) M1-phenotype. The functional endpoint is the capacity of MSC-exosomes to suppress the mRNA induction of TNFα. The half maximal effective concentration (EC_50_ value, 50% inhibition in TNFα mRNA levels, relative to M1 control) is transformed to an arbitrary EPU (described in Figure 4) (8). In turn, an EPU could potentially be applied to standardize dosing between different exosome preparations. In all cases, potency assays need to be disease specific, fit-for-purpose, and employ relevant functional end-points.

## Manufacturing and Scale-Up

In 2002, Lamparski et al. described a method for the production and characterization of clinical grade exosomes derived from dendritic cells for their application in cancer vaccine clinical trials (85). Using ultrafiltration coupled with a sucrose/deuterium oxide UC cushion, they isolated vesicles (50–90 nm in diameter) containing major histocompatibility complex (MHC) class-I, -II, and CD1, and tetraspanin molecules (CD9, CD63, and CD81) In addition, in 2005, Navabi and coworkers detailed the development of a method for the preparation and characterization of good manufacturing practice (GMP)-grade exosomes from the ascites fluid of ovarian cancer patients (86). Since then our understanding of exosome biology has improved and the development of specialized isolation and characterization methods has allowed investigators to more accurately isolate and characterize exosome populations.

Considering the development of exosome-based therapeutics, lessons could be learned from cell therapy. The *in vitro* expansion of cells (such as MSCs) is required to deliver an effective therapeutic dose, with the absence of having a detrimental impact on the quality of the cell. Upon scaling-up, process analytical technology (PAT), a system proposed by the FDA, may be implemented to monitor the manufacturing process through continuous measurement of cell parameters (87, 88). Monitoring bioprocess parameters, such as population doubling time, temperature, metabolite concentrations, pH, *p*O_2_ and *p*CO_2_, may help ensure optimal exosome quality and quantity, as previous reports have shown that subtle acidic pH shifts may impair exosome aggregation inhibiting forces and, in turn, promote aggregation and reduce functionality (56).

Recently, the inevitable shift to using tissue culture bioreactors has been used to generate large-scale EV preparations. Indeed, Watson and colleagues demonstrated that hollow-fiber bioreactors promotes enhanced exosome production (~40-fold greater EVs/ml of CM) when compared to conventional 2D tissue culture preparations (89). However, it remains unclear if such methods enhance generalized EV production or simply reflect a reduction in exosome re-uptake. Under the considerations discussed above, relating to EV diversity and the possibility that only specific exosome subtypes may represent the therapeutic agent, it is premature to assume that production of higher EV numbers will necessarily yield a higher efficacy final product. Optimization of MSC culture conditions will, therefore, require the parallel development of a dependable and easily adoptable potency assay.

## “Off-The-Shelf” Exosomes

With evidence to suggest that exosomes can be stored at −20°C for up to 6 months with no loss to their biochemical activity, “off-the-shelf” exosome-based products represent an attractive pharmaceutical formulation (56, 66, 90). Although standardized storage procedures remain to be defined, current storage protocols use isotonic buffers to prevent pH shifts during storage, avoid freeze–thaw cycles and are absent of dimethyl sulfoxide (DMSO) and glycerol as previous reports have shown these agents may impact exosome integrity (91). With a lack of data addressing the impact of storage time and excipients on exosome structural stability and functional efficacy, more studies are warranted to help define a provisional “shelf-life” for exosome-based products and facilitate the manufacturing and distribution process.

## EV-Based Therapy in Clinical Trials

Promising preclinical data that demonstrated dendritic cell-derived EVs containing MHC–peptide complexes could alter tumor growth in immune competent mice led to a phase I anti-melanoma clinical trial conducted in France (92) and a phase I anti-non-small cell lung cancer clinical trial in the United States (93) (clinical trial applications highlighted in Table 4). Both clinical trials administered autologous dendritic cell-derived EVs that met their respective current GMP standards. Such clinical trials to date are important for their demonstration of both the feasibility and the short-term safety of autologous EV administration, but safety considerations for therapies based on exogenous exosome-based products will arguably be more stringent. Nevertheless, it is very encouraging to note that preclinical studies have established immunomodulation as the main therapeutic mechanism of MSC-exosomes action. Immunomodulation is clearly involved in the autologous exosome clinical trials mentioned above, and this may provide guidelines and precedent for clinical trials using exogenous exosomes. In this context, a recent clinical case involving treatment of a steroid-refractory graft-vs-host disease patient with MSC-EVs derived from unrelated bone marrow donors produced encouraging results (94).

**Table 4:** Exosome-based therapy: clinical trials.

Disease	Phase	Vesicle cellular source	Route of administration	Isolation method	Modified (Y/N)	Status	Reference
Melanoma	I—open label	Autologous monocyte-derived dendritic cells	SC	UF/UC sucrose cushion	Y	Complete	Escudier et al. (92)
Non-small cell lung cancer	I—open label	Autologous monocyte-derived dendritic cells	SC and intradermal	UF/UC sucrose cushion	Y	Complete	Morse et al. (93)
Colon cancer	I—open label	Autologous ascites	SC	UC sucrose cushion	N	Complete	Dai et al. (95)
Colon cancer	I—open label	Plant based	–	Not declared	Y	Ongoing	NCT01294072
Type I diabetes	I—open label	Umbilical cord blood (allogeneic) MSC	–	Not declared	N	Ongoing	NCT02138331
Non-small cell lung cancer	II—open label	Tumor cell	Pleural or peritoneal cavity	Not declared	Y	Complete	Besse et al. (96)
Wound healing (Ulcer)	I—open label	Plasma (autologous)	–	Not declared	N	Enrolling	NCT02565264

*MSC, mesenchymal stem/stromal cells; SC, subcutaneous; UF, ultrafiltration; UC, ultracentrifugation*.

*Adapted from Lener et al. (56)*.

**Figure 5:**
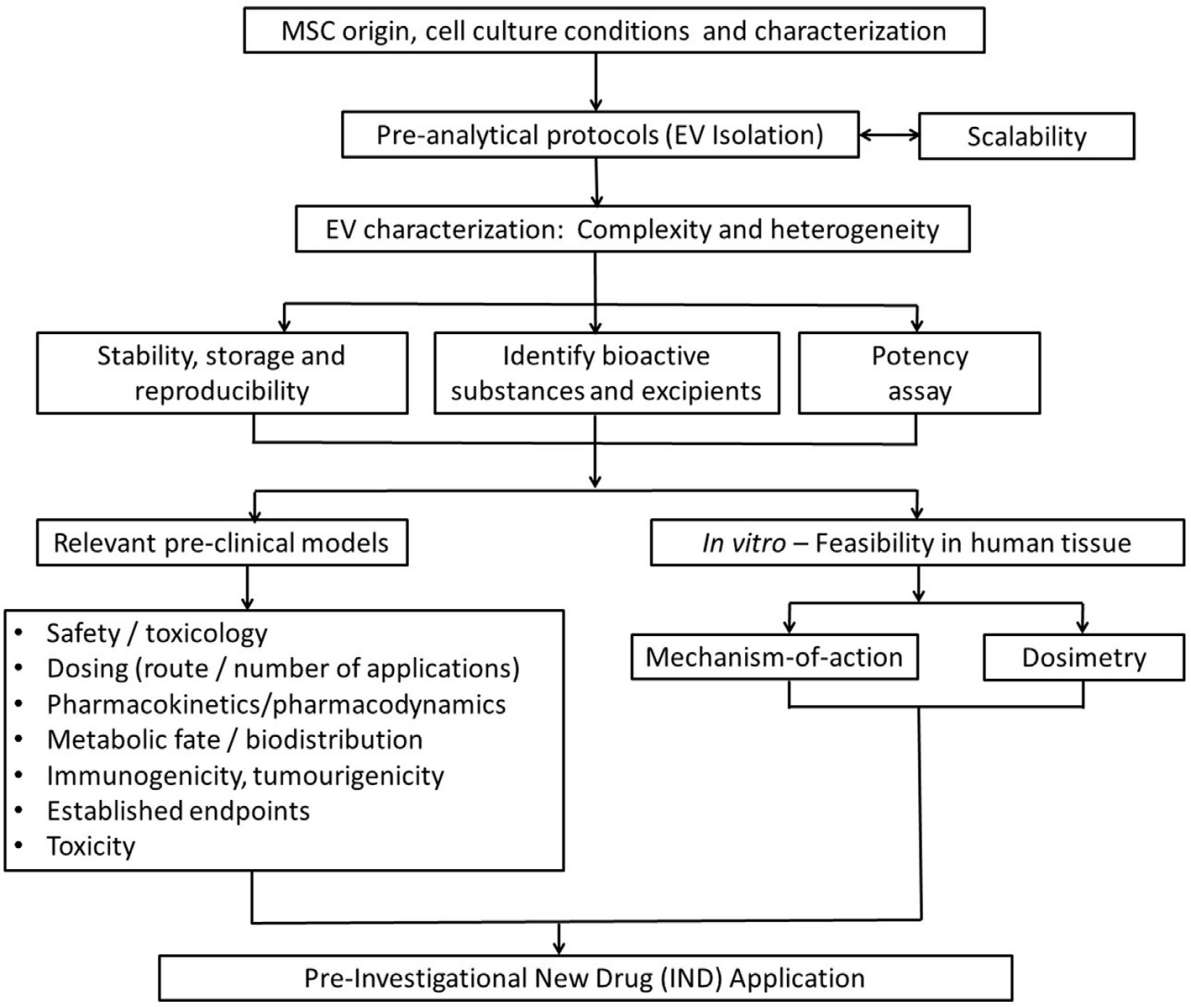
Strategic flowchart for the preclinical testing of exosome-based therapeutics. EV, extracellular vesicle. Adapted from Ref. (97).

Ultimately, issues raised in this review aim to provide a basic guidance for investigators on key issues to consider for the smooth transition of exosome-based therapies from the preclinical model into clinical development (Figure 5). Among them, determining the optimal dose, the appropriate time window for exosome administration, the number of doses, and route of administration that achieves maximal efficacy without adverse effects are the most important issues to resolve. Such issues will be disease/model specific and clearly beyond the scope of this work.

## Summary

Exosome-based therapeutics represent a most promising next generation approach for treating a diverse number of diseases, particularly diseases the pathogenesis of which involves a primary (or major) inflammatory component. The efficacy of MSC-exosome treatments has been robustly established in numerous preclinical models, but development of large-scale GMP-grade exosome-based pharmaceuticals and subsequent clinical trials demand the resolution of several technological and mechanistic issues, reflecting the cautious navigation in unknown seas for this relatively novel field. Among the major issues to be resolved are the definition of an EPU, the standardization of MSC culture conditions and protocols for exosome harvest and storage. Although safety considerations need also to be addressed, it is expected that safety concerns for cell-free, exosome-based clinical trials will be arguably milder than those relevant to live cell MSC trials currently in progress, as mutagenicity and oncogenicity concerns will be null.

## Author Contributions

GW participated in data collection, analysis, and manuscript writing. SK and SAM contributed to final article editing and approval.

## Conflict of Interest Statement

The authors declare that the research was conducted in the absence of any commercial or financial relationships that could be construed as a potential conflict of interest.
